# Carrots for the donkey: Influence of evaluative conditioning and training on self-paced exercise intensity and delay discounting of exercise in healthy adults

**DOI:** 10.1371/journal.pone.0257953

**Published:** 2021-10-06

**Authors:** Hans-Peter Kubis, Tamam A. Albelwi, Robert D. Rogers

**Affiliations:** 1 School of Sport, Health and Exercise Sciences, Bangor University, Bangor, Wales, United Kingdom; 2 Ministry of Health Saudi Arabia, Arar, Northern Border Zone, Saudi Arabia; 3 School of Psychology, Bangor University, Bangor, Wales, United Kingdom; University of Essex, UNITED KINGDOM

## Abstract

To choose exercise over alternative behaviours, subjective reward evaluation of the potential choices is a principal step in decision making. However, the selection of exercise intensity might integrate acute visceral responses (i.e. pleasant or unpleasant feelings) and motives related to goals (i.e. enjoyment, competition, health). To understand the factors determining the selection of exercise in its intensity and evaluation as a modality, we conducted a study combining exercise training and evaluative conditioning. Evaluative conditioning was performed by using a novel technique using a primary reinforcer (sweetness) as the unconditioned stimulus and physical strain i.e. heart rate elevation as the conditioned stimulus during interval training, using a randomized control design (N = 58). Pre, post-three weeks interval training w/o conditioning, and after 4 weeks follow-up, participants were tested on self-paced speed selection on treadmill measuring heart rate, subjective pleasantness, and effort levels, as well as delay-discounting of exercise and food rewards. Results revealed that the selection of exercise intensity was significantly increased by adaptation to training and evaluative conditioning, revealing the importance of visceral factors as well as learned expected rewards. Delay discounting rates of self-paced exercise were transiently reduced by training but not affected by evaluative conditioning. In conclusion, exercise decisions are suggested to separate the decision-making process into a modality-specific cognitive evaluation of exercise, and an exercise intensity selection based on acute visceral experience integrating effort, pleasantness, and learned rewards.

## Introduction

Exercise and physical activity are strongly associated with physical and mental wellbeing [1–3], but only a small proportion of the population meets the required recommendations in physical activity [4–6]. Additionally, exercise with higher intensities is found to be more beneficial for health protection than lower intensities [7, 8]. Certainly, an individual who decides to take part in exercise evaluates the modality options (exercise versus others), but when having decided to participate in an exercise, also of what intensity and duration. Consequently, the decision-making process entails at least two main steps, a modality choice, and an exercise intensity choice, which may be influenced by different processes and factors. Besides many environmental and personal factors determining physical activity [9], motivation with its intrinsic and extrinsic components are known to be critical for exercise participation (i.e. frequency) [10]. Extrinsic motives may be related, as an example, to delayed outcomes like health and looks [11, 12]; however, intrinsic motives can contain components, which are derived from pleasure and satisfaction of engaging in a behaviour [13]. While pleasure and satisfaction of taking part in exercise could have numerous facets, like being competitive or enjoying social interaction, less is known about the rewarding properties of exercise in itself as a physical strain [14]. Several studies have shown that high intensities can be perceived as pleasant [15–17], and affective response to a bout of exercise could predict physical activity over 6 and 12 months [18]; however, self-determined intensities are more preferred over externally controlled ones [19–21]. On the other hand, behavioural models of effort discounting, where a person needs to put effort into a task to gain an external reward, revealed that people try to maximize gain while effort is minimized [22, 23]. Effort commonly carries a negative value or cost, which provides a reference against which rewards are evaluated; a reward is higher in subjective value if it is earned with easier than greater effort [24, 25]. This concept is supported in humans, as well as in animal studies [25–30]. Indeed, this concept can only be applied to exercise as such, if we assume that exercise has an inherent rewarding property related to physical strain.

We have recently investigated the question of a potential reward value of exercise by investigating the delay discounting of self-selected exercise on treadmill [31]. Delay discounting poses choice questions of immediate or delayed rewards with variable reward and delay magnitude [32, 33]. People tend to discount rewards over time that immediate smaller rewards are preferred over delayed larger ones; rewards lose subjective value with waiting time until receipt. Our former study showed that self-selected exercise was discounted in time like other ‘consumable’ rewards (i.e. food) and that the rate of delay discounting was negatively associated with exercise motivation, as well as being reduced by training [31]. Slower discounting has been assumed to be a sign of stronger involvement of cognitive elaborative processing over impulsive decision making [34]. However, the effects of training selection of exercise intensity (increase) and on discounting (reduction) could not be explained by a unifying process. According to Loewenstein [35], the defining characteristic of a visceral (e.g. pleasure, discomfort, etc.) factor is a direct hedonic impact and effect on the desirability of goods and actions. This certainly open the questions, how the potential rewarding properties of exercise are balanced with its effortful components, and whether we can manipulate those properties inducing preference for higher intensities? Also, does alteration of these visceral properties affect the cognitive evaluation of exercise i.e. delay-discounting? The visceral properties of exercise during self-selection of exercise intensity could be potentially influenced by training altering physiological response to exercise, and via association of a rewarding stimulus with increased physiological strain. Evaluative conditioning (EC) can be defined as the procedure which changes the valence of a stimulus (conditioned stimulus–CS) that is induced by the pairing of that stimulus with another positive or negative stimulus (unconditioned stimulus–UCS) [36, 37]. EC concerns only evaluative responses to the conditional stimulus, therefore influencing only its liking rather than a change in the type of response being expected from Pavlovian conditioning [37]. Moreover, EC is known to produce stable effects in various paradigms using visual and appetitive stimuli as unconditional stimuli [38–40]. In the context of physical activity, only a few studies performed EC using visual stimuli for conditioning [41, 42]. However, sweet rewards are often used in animal conditioning paradigms [43–45] but less in humans [39, 40, 46]. The rewarding nature of sweetness and the strength of its addictive potential are confirmed numerous times [43, 44, 47], and, even non-caloric sweeteners have the potential to be used in conditioning paradigms [43, 48].

In this study, we used a novel evaluative conditioning (EC) paradigm, pairing a primary reinforcer (sweet solution) with cardiovascular strain during exercise interval training over three weeks. A further training-only group received a neutral saline solution during trainingand a control group received no training and no conditioning. Pre and post, as well as 4 weeks after training (follow-up) subjects performed sessions for self-selected speed selection and delay discounting of the selected exercise; subjects selected intensity of exercise to adjust to maximize pleasantness (Feeling Scale) and also reported rate of perceived exertion/effort (RPE); besides, heart rate, body characteristics, and a battery of psychological questionnaires plus an assessment of delay discounting rate of the self-selected exercise, favorite food and money were performed with a computer paradigm [31].

We hypothesized that evaluative conditioning would increase the self-selected speed with a concomitant increase in heart rate and RPE levels after training and follow-up above the level induced by training only, assuming that the conditioned reward would be integrated into the exercise reward. Exercise training alone would lead to a transient increase of self-selected speed due to transient physiological adaptations and a decline at the follow-up; training adaptations would lead to changes in physiological strain perceived as rewarding.

Moreover, the elevation of the reward value after training and conditioning would induce a reduction of delay discounting of exercise due to a magnitude effect [49].

## Materials and methods

### Participants

After ethical approval by the ethics committee of the School of Sport, Health and Exercise Science, Bangor University (ethics number: P05-16/17), 62 subjects (32 females) were recruited from students and the general public in Bangor, UK, and 58 finished the study; two participants dropped out without stating reasons, two were excluded because of missing training sessions. Eligible for participation were healthy female and male subjects aged between 18–50, who did not engage in regular physical activity or dieting. Subjects with medical conditions that contraindicated performing regular high-intensity interval training were excluded. Participants received £100 as a reimbursement for their time. The sample size was calculated based on the study by Antoniewicz and Brand [42], who used EC with visual stimuli and observed an increase in exercise intensity selection. The power analysis aimed to detect a significant difference in exercise intensities between groups using G*Power 3.1.9.2, ANOVA: Repeated Measures, within-between interactions at a significance level of 5%; a sample size of 16 (8 in each group) would have 95% power to detect an effect based on a partial eta squared of 0.290 between groups. The sample size for the effect of exercise training on discounting rates, based on Albelwi et al. [31], aimed to detect a significant difference between groups, ANOVA: Fixed effects, special, main effects and interactions at a significance level of 5%; a sample size of 54 (divided into 2 groups) would have 90% power to detect an effect size of 0.204 between groups.

### Design

**Fig 1:**
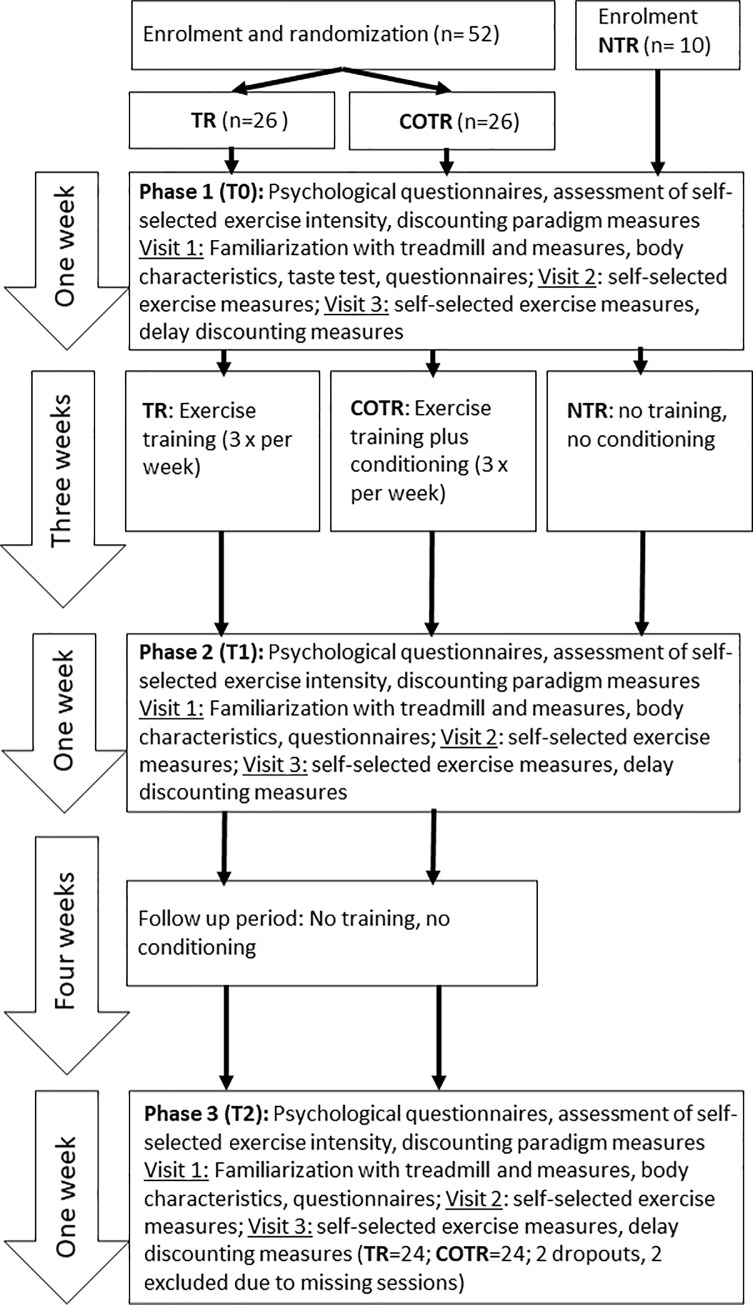
Flow diagram of the intervention through the phases baseline (T0), post-training (T1), and follow-up (T2).

A between- and within-subjects experimental design was used investigating self-selected exercise intensity, heart rate, RPE, as well as delay discounting of exercise over the three study phases, baseline, post-training, and follow-up. Fifty-two participants were randomly assigned into two groups, a training group (TR) who received unflavoured electrolyte mouth rinse, and a conditioning plus training group with sweet mouth rinse group (COTR) during all interval training sessions. A no training, no conditioning group (NTR) was recruited separately from the same population for testing of training effects on parameters (n = 10) (Fig 1).

### Physical characteristics and physiological parameters

Weight and body composition (i.e. percent body fat) were assessed via bioelectrical impedance measurement using a Tanita BC-418 MA system. Participants’ height was measured using a standard stadiometer. Heart rate was measured during all exercise sessions (self-selected exercise sessions and interval training sessions) using a Polar heart rate monitor in connection with a bespoke computerized system for EC.

### Self-report measures

Participants were asked to fill out selected questionnaires for better characterization of the population sample and to measure exercise motivation (full list and details, see S1 File).

1. International Physical Activity Questionnaire (IPAQ) [50] is a standardised self-report measure of habitual physical activity. Reliability was tested over 12 countries: Spearman’s rho 0.81 [51].

Exercise Motivation Inventory (EMI 2) [52] assesses exercise participation motives applicable to both exercisers and non-exercisers. We used the items challenge, affiliation, revitalization, and enjoyment as intrinsic factors (this study, Crα: 0.788), and appearance, weight management, positive health, health pressure, ill-health avoidance, and strength/endurance as extrinsic factors (this study, Crα: 0.713), according to Egli [12]. Further motives (e.g., social recognition, stress management) are more difficult to classify along with dichotomous categories and were included in the total exercise motivation score (this study, Crα: 0.883).

### Procedure: Phase 1: Baseline measurements of self-selected exercise

#### Visit 1

Participants were informed to wear comfortable clothing that allows exercising on all visits. In addition, participants had to abstain from alcohol and caffeine consumption for at least 12 hours, and not engage in strenuous exercise for at least 48 hours. Visits were scheduled at consistent times for the individuals (morning/afternoon). They were introduced to the protocol, consent was given, and asked to fill out self-report questionnaires (see self-reported measures), followed by measuring body characteristics. Then, participants were asked to walk for about 3 to 5 minutes on the treadmill to be familiarised with the exercise and manual settings of the treadmill for further visits.

The goal for the exercise trials (1st and 2^nd^ visits) was to establish the most pleasant/enjoyable exercise intensity possible for each participant to establish. This specific exercise experience would later serve as the exercise imagined during the delay discounting task. The exercise intensity was set by repetitively self-adjusting the treadmill speed during the trials (see below). Social desirability and demand characteristics were minimized by emphasizing the aim to find the optimal exercise intensity for the participant’s enjoyment using the same verbal protocol for all assessments and involving five different experimenters for reduction of bias and interpersonal contact.

All trials were performed using the same treadmill; during the exercise period, a nature soundtrack consisting of bird and forest sounds was played through speakers while the participants were facing a natural scenery through a wide window. This was performed to reduce possible negative effects of the technical environment on participants’ perception. The heart rate (HR) was monitored throughout and after the end of the exercise sessions with a HR monitor (Polar RS800CX). Exercise trials were terminated after 30 minutes or whenever the participant chose to end it earlier. The sessions were separated by at least 48 hours and maximally by one week. Before each exercise trial, each participant warmed up on a cycle ergometer (Lode Excalibur) for 3 minutes before stepping on the treadmill.

For the first exercise trial, participants started walking on the treadmill at 3 km/h; display of speed and time was concealed from individuals in all trials. They were instructed to find the most pleasant exercise intensity they could adjust by modifying the speed of the treadmill using the control panel; it was emphasized that the experiment was not about fitness or performance. Participants were told that the exercise duration was up to 30 minutes maximally. After 2 minutes of exercise, participants were asked to rate the pleasantness of exercise using the 11-point Likert Feeling Scale (FS) that ranges from -5 to ‏5; anchors are provided at zero (‘Neutral’) and all odd integers, ranging from ‘Very Good’ (‏5) to ‘Very Bad’ (-5) [53]. For the rating, the participants were asked ‘How do you rate the current exercise of being pleasant?’. Participants could modify the treadmill speed every two minutes to optimise pleasantness, e.g. increasing or decreasing the speed (selection could be made during the first 30 sec of the two minutes). Rating of current pleasantness was requested after the two-minute period elapsed; any set values were not visible for participants. After cooling down, the participants were free to leave.

#### Visit 2

The second exercise trial had the goal to reconfirm the setting and experience of the self-selected exercise. After warming up, participants walked on the treadmill at 3 km/h and the speed was elevated gradually to the preferred speed selected in the first exercise trial by the experimenter and held for further 2 minutes. The researcher then manipulated the speed by increasing and decreasing it by 10% of the preferred speed level, each period for 2 minutes, while pleasantness was rated every 2 minutes to confirm the optimal setting of the preferred speed [54]. Thereafter, a 5 minutes rest was given, and participants performed a further 5–10 minutes bout at the preferred speed to validate perception and to reinforce the feeling regarding this exercise bout.

#### Visit 3

The purpose of this exercise trial was to explore the perception of perceived exertion/effort at preferred exercise speed. This measure was not introduced during the previous two trials to avoid any cross-over effects between the two perceptual modalities (pleasantness and effort). However, the RPE scale was thoroughly explained verbally and anchored by memory. After the warmup, participants started walking on the treadmill, and then the speed was gradually increased to the formerly self-selected preferred exercise speed by the experimenter; the speed was increased, as well as decreased, according to the protocol of visit 2. Participants were asked to rate their perception of exertion/effort every 2 minutes using Rate of Perceived Exertion (RPE) scale [55] which starts from 6 (no exertion at all) to 20 (maximal exertion). Thereafter, a 5 minutes rest was given, and participants performed a further 5–10 minutes bout at the preferred speed to validate effort ratings. RPE values were taken from the periods at self-selected speed and from the bout after rest; the most frequent value of RPE at self-selected speed was used.

After a resting period of about 5 minutes, participants were introduced to the delay discounting task on the computer.

#### Delay Discounting (DD) tasks

Tests were performed according to Albelwi [31]; in brief, each participant was verbally introduced to the task, read the introductions, and followed instructions on the computer screen. The DD tasks were generated using a specially designed computer programme based on the paradigm described by [56] via the Inquisit™ program (developed by Milliseconds Software). The indifference points (IP) for each time delay of rewards for the tested commodities were obtained by randomization between delays and the size of rewards. The sooner, the smaller hypothetical reward was offered ‘at the end of this session’ as an immediate choice, and after 1, 7, 30, 60, and 180 days delay. The values of the three commodity rewards were adjusted based on their monetary value [57, 58]. The adjustment of the rewards was masked by randomization between delays and amount of rewards, and with the progression of the test, distractors were displayed to prevent the subject from predicting the questions and unmasking the underlying technique of the test as recommended by [56]. The program terminated automatically and saved the experimental data after IP criteria had been achieved. Each computer task took about 4–6 minutes to be finished.

*Money DD task*. For the monetary rewards, the hypothetical amounts offered were (£2-£7-£12-£17-£22-£27-£32-£37-£42-£47 and £50). The script for this task can be found in the S1 File.

*Food DD task*. For the reward of food, the hypothetical amounts offered were 5, 10, 15, 20, and 30 bites (1 plate); 60 bites (2 plates), 90 bites (3 plates), 120 bites (4 plates), and 150 bites (5 plates) of food as the largest reward. The complete script can be found in the S1 File.

*Exercise DD task*. For the exercise, the hypothetical exercises sessions offered were based on the formerly established treadmill exercise sessions (see above) and were fragmented into (5, 10, 15, 20, and 30 minutes (1 gym session), 60 minutes (2 gym sessions), 90 minutes (3 gym sessions), 120 minutes (4 gym sessions) and 150 minutes (5 gym sessions); assuming that 1 gym session = 30 minutes of exercise. The complete script is to be found in the S1 File.

The taste test was administered by the end of this phase for the COTR subjects (see S1 File).

### Phase 2: Interval training sessions with and without evaluative conditioning

#### Evaluative conditioning paradigm

*Syringe pump system*. Two 60 ml syringes filled with either (2 x 60 ml NEUTRAL SOLUTION (see S1 File) for the TR group, or 1 x 60 ml NEUTRAL SOLUTION and 1 x 60 ml 100% SWEET SOLUTION (see S1 File) for the COTR group) were attached to two New Era programmable syringe pumps, Model: NE-4000. The combined flow rate was 2 ml/minute infused into a double tubing system and released through a double-barrel mouthpiece onto the participant’s tongue/oral cavity. The selected flow rate of 2ml/min is in the range of normal stimulated saliva flow rate in adults which is 1–3 mL/minute [59] to minimize swallowing during exercise. The TR group subjects received a constant NEUTRAL SOLUTION injection into the mouth during interval training, COTR subjects received NEUTRAL SOLUTION with SWEET SOLUTION admixture depending on HR. The admixture was controlled by a bespoke computer program using the HR measure for adjusting sweetness of mouth rinse solution of the COTR group ≤ 85% of calculated HR max received 100% SWEET SOLUTION, while HR at individually self-selected speed (baseline) received 0% sweetness = NEUTRAL SOLUTION. While the total liquid rate kept constant (2ml/min), any increase in HR above baseline increased the sweetness admixture in a quadratic exponential manner achieving 100% sweetness at 85% HR max, see Eq 1.


$$sweet\;solution\;rate=\frac{(current\;HR-baseline\;HR)2}{(85\%\;HRmax-baseline\;HR)2}*total\;liquid\;rate$$ 1)

The pairing between heart rate change (conditioned stimulus, CS) and the sweet reward (unconditioned stimulus, US) depended on exercise intensity increments during interval training. Six pairing periods were applied per training session; a total of 54 pairings over 9 conditioning sessions were applied during interval training sessions over 3 weeks [60]. Pairings were induced during the interval training sessions in the COTR groups, while the TR group received the same interval training with NEUTRAL SOLUTION injection. All participants attended all nine interval training sessions over three weeks.

#### Interval training sessions

The interval exercise training consisted of three sessions per week over three weeks for TR and COTR groups. The exercise training was performed using an interval training protocol on a treadmill consisting of progressive peak training intensities between 60–85% of the estimated HR_max_. HR_max_ was calculated via 220 –age = HR_max_ which is suitable for the recruited age group [61]. Target velocities for the treadmill were calculated by using the HR/treadmill speed relationship from the assessment trials for self-selected speed. Speed was adjusted manually by the researcher if the target HR was not achieved. Subjects were verbally informed about oncoming increases or decreases in speed to avoid accidents. The training protocol was identical for TR and COTR groups.

After a warm-up on a cycle ergometer (Lode Excalibur) for 3 minutes, subjects started exercising on a treadmill (computer-controlled treadmill, h/p-Cosmos) and gradually increased to participants’ preferred speed (baseline). Subjects were blinded to all data of the treadmill settings. This was followed by intervals ramped from baseline to 60% HRmax over ~10 sec, then held for 2 minutes, followed by slowing down to baseline speed over ~10 sec; baseline speed was then held for further 1.5 minutes followed by the next cycle increasing intensity by 5% of HRmax. Six cycles were performed per training session (i.e. 60%, 65%, 70%, 75%, 80%, 85% of HRmax), followed by a cool-down at walking speed. Total exercise time was about 30 minutes.

### Phase 3: Post-training assessments

It included 3 sessions, carried out during the following week after the exercise interval training phase for TR and COTR groups, for the control group (NTR), three weeks after phase 1 (no training). All sessions and measurements were performed in the same order and ways as described for baseline measurements except omitting the taste test.

### Phase 4: Follow-up assessments

These were carried out 4 weeks after post-training assessments (phase 3) for TR and COTR groups. During this period participants were instructed not to engage in any physical training that was out of their usual former (before the intervention) daily routine. This phase included three sessions, using the same protocol as for phase 3. Subjects were reimbursed after this session and debriefed; participants were initially informed that the study was aimed to investigate the influence of oral cavity rinse to avoid dry mouth during the exercise.

### Analysis

All variables were tested on assumptions for parametric testing (i.e. mixed model ANOVA and ANCOVA, t-test); parameters, which were not normally distributed were transformed (by log: delay discounting constants k, speed, heart rate; X^2^: rate of perceived exertion (RPE)) to comply with ANOVA/ANCOVA test assumptions. ANOVA and ANCOVA were applied according to recommendations by Van Breukelen [62] with Bonferroni correction. Parameters, which could not be successfully transformed were analyzed using non-parametric analysis e.g. Kruskal-Wallis followed by Mann & Whitney U-tests as indicated in the results section. Multiple regression analysis was performed using the enter method of selected parameters, as indicated in the results section. For model fitting of the hyperbolic effort discounting equation [63] on selected data, the Microsoft Excel Solver program using the least square fit method was used to obtain effort discounting constant value k. The fit of model parameters was tested using the Wilcoxon sign test. Besides, delay discounting constants k for money, food, and exercise were calculated using Mazur’s equation [64] fitting participants’ indifference points (IP) data to hyperbolic functions using the least square fit method with the Microsoft Excel Solver program. Data sets were removed if poor-fit in the hyperbolic model (R^2^ <0.7). Correlation analysis was performed using Spearman’s and Pearson’s correlation analysis. Data are displayed in mean and standard deviation, or median, and 25 and 75 percentiles. Significance levels were reported if lower than p<0.05. Data were analyzed using Statistical Package for the Social Science (IBM SPSS) version 25.

## Results

### Physiological and psychological characteristics

58 participants, out of 62 recruited, completed the study (32 females). 48 participants concluded the randomized control trial, training group (TR) (n = 24) and training plus conditioning group (COTR) (n = 24); in the no-training group (NTR), to control for time effects over the training period, ten participants completed (n = 10). Body characteristics and psychological self-report parameters are shown in Table 1 and S1 Table in S1 File in supplements. Participants were young adults (24.3 (5.2) yrs.), with a wide range of BMI (BMI 18.5–40.5) but mostly eutrophic (53%), mainly reported moderate to high physical activity (86%), and more than medium exercise motivation (EMI 2). Participants were not aware of the EC process; only 3 out of 24 participants of the COTR group reported contingency awareness of higher speed with sweetness after the intervention (debriefing). Concurrently, we assume that any conditioning effects were produced out of awareness.

**Table 1:** Mean and standard deviations of body characteristics and psychometric self-reports.

	No Training (NTR) (N = 10)	Training (TR) (N = 24)	Training plus conditioning (COTR) (N = 24)
Sex	Female 70%	Female 50%	Female 54%
Age (yrs)	26.00 (5.12)	23.67 (4.19)	24.25 (6.05)
Weight (kg)	64.18 (8.69)	69.68 (15.92)	78.49 (19.36)
BMI (kg/m^2^)	23.46 (3.24)	24.78 (4.61)	27.06 (5.40)
PCT of body fat (%)	24.74 (8.85)	23.55 (9.33)	26.65 (9.66)
IPAQ	h = 6; m = 3; l = 1	h = 5; m = 15; l = 4	h = 10; m = 11; l = 3
EMI 2 (RG: 0–5)			
Extr. Ex. Mot.	3.60 (0.53)	3.28 (0.75)	3.30 (0.63)
Intr. Ex. Mot.	3.18 (1.15)	3.20 (0.89)	3.35 (0.38)
Intr. plus Extr. Mot.	2.84 (1.31)	2.42 (0.85)	2.62 (0.95)

RG, range of scores; PCT, percentage; h, high; m, medium; l, low; Intr., intrinsic; Extr., extrinsic; Mot., motivation.

### Effects of training and conditioning on self-selected speed, heart rate, and RPE

Training improved participants’ cardiovascular exercise efficiency; heart rate per speed (HR/Speed) (Table 2) was significantly reduced after training in both TR and COTR groups (mixed model ANOVA; main effect of time: F = 21.87, p<0.0001, η^2^ = 0.504; contrast baseline versus post-training: F = 43.48, p<0.0001, η^2^ = 0.497; no significant interaction of group x time); no changes were detected in the NTR group.

**Table 2:** Exercise trial characteristics for no training (NTR), training (TR), and Conditioning Training (COTR) groups.

Groups	No Training (NTR) (N = 10)		Training (TR) (N = 24)		Training plus conditioning (COTR) (N = 24)	
Parameters	Mean STD (±)	MED., (25^th^| 75^th^ PCTL.)	Mean STD (±)	MED., (25^th^| 75^th^ PCTL.)	Mean STD (±)	MED., (25^th^| 75^th^ PCTL.)
Preferred Speed (km/h)—T_0_	6.03 (2.34)	5.70 (4.70|6.25)	5.25 (1.27)	4.80 (4.30|5.90)	5.71 (1.22)	5.40 (4.93|6.40)
Preferred Speed (km/h)—T_1_	6.06 (2.48) †	5.65 (4.73|6.33)	6.29 (1.47)* †	5.80 (5.60|6.80)	7.01 (1.56)*	6.45 (5.95|7.27)
Preferred Speed (km/h)–T_2_	N/A	N/A	5.42 (1.02)#	5.27 (4.76|5.77)	6.53 (1.47) #	6.33 (5.80|7.27)
Avg. HR (bpm)—T_0_	120.4 (20.5)	118.5 (102.5|134.5)	118.7 (15.3)	118 (107.0|124.0)	123.1 (21.6)	117.5 (108.3|137.0)
Avg. HR (bpm)—T_1_	120.5 (20.6) †	117.5 (101.8|132.5)	127.3 (17.1)* †	125.5 (114.0|136.0)	135.5 (21.1)*	134.5 (115.5|154.3)
Avg. HR (bpm)–T_2_			119.0 (10.9) #	117.0 (111.5|125.5)	130.5 (20.2) #	131.0 (116.0|143.5)
HR/Speed (bpm/km*h^-1^)–T_0_	21.14 (4.00)	21.07 (19.40|22.65)	23.21 (4.03)	23.98 (19.10|26.62)	21.94 (3.44)	22.07 (18.79|24.31)
HR/Speed (bpm/km*h^-1^)–T_1_	21.11 (3.71)	21.61 (19.54|22.72)	21.28 (3.05)*	21.58 (18.36|23.38)	19.69 (2.59)*	19.34 (17.84|21.74)
HR/Speed (bpm/km*h^-1^)–T_2_	N/A	N/A	22.81 (3.25)	23.42 (20.47|25.04)	20.41 (2.90)	20.07 (18.49|22.20)
RPE (RG: 6–20)—T_0_	10.30 (2.45)	10.50 (8.00|11.50)	9.83 (1.98)	10.00 (8.25|11.00)	9.82 (1.89)	10.00 (9.00|11.00)
RPE (RG: 6–20)—T_1_	10.50 (2.51)	10.00 (9.00|12.00)	10.63 (1.76)* #	11.00 (9.00|12.00)	11.46 (1.37) #	12.00 (11.00|12.00)
RPE (RG: 6–20)–T_2_			10.13 (1.78) #	10.00 (8.25|11.00)	10.86 (1.61) #	11.00 (10.00|12.00)
Feeling Scale (RG:-5/0/5)—T_0_	4.00 (1.16)	4.50 (3.00|5.00)	4.25 (0.99)	5.00 (3.00|5.00)	4.29 (0.96)	5.00 (3.25|5.00)
Feeling Scale (RG:-5/0/5)—T_1_	3.90 (1.10)	4.00 (3.00|4.00)	4.25 (0.94)	5.00 (3.25|5.00)	4.42 (0.88)	5.00 (4.00|5.00)
Feeling Scale (RG:-5/0/5)–T_2_	N/A	N/A	4.21 (0.83)	4.00 (3.25|5.00)	4.33 (0.82)	5.00 (4.00|5.00)

*Significant effect of training, p<0.05

#significant interaction of group x time (TR, COTR), p<0.05

†significant interaction of group x time (NTR, TR), p<0.05. MED, median; PCTL, percentiles; STD, standard deviation; RG, range of scores; Avg., average; HR. heart rate; RPE, rate of perceived exertion/effort.

**Fig 2:**
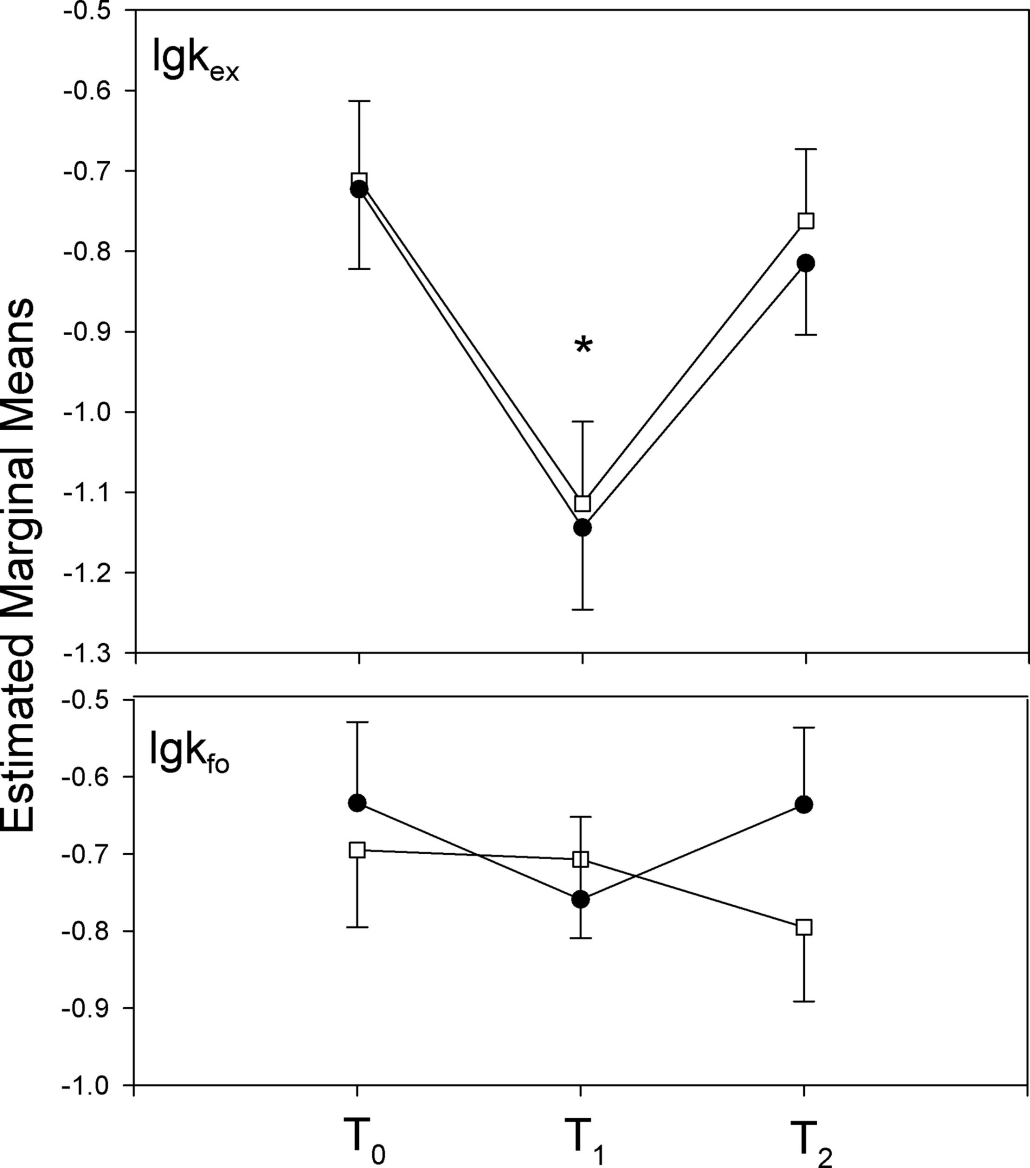
Log-transformed speed, Heart Rate (HR), and Rate of Perceived Exertion (RPE) change over the three time points; baseline (T_0)_- post-training (T_1_) and follow up (T_2_) for training (TR) and Conditioning Training (COTR) groups. Open boxes COTR; closed boxes TR. *Significant effect of training, p<0.05; #significant interaction of group x time; the figure shows means and SE. Data are depicted and used in the form of meeting statistical test assumptions. Untransformed data are presented in Table 2.

Analyzing the self-selected speed, heart rate, and RPE data of the randomized control trial, results show that self-selected speed was selected on significantly higher levels after training than at baseline in both, TR and COTR groups. A main effect of time was reported in the ANCOVA with baseline speed as a covariate (F = 6.65, df = 2, p = 0.003, η^2^ = 0.236), contrasts detected a significant increase in speed at post-training (T_0_ versus T_1_: F = 9.64, df = 1, p = 0.003, η^2^ = 0.180). The self-selected speed was about 1 km/h faster after training than at baseline in both groups (see Table 2); no significant interaction of group x time was reported between baseline (T0) and post-training (T1). ANCOVA, however, reported a significant interaction of group x time (F = 7.70, df = 2, p = 0.001, η^2^ = 0.264), whereby contrasts revealed that the significant interaction was between after training (T_1_) and 4 weeks follow-up (T_2_), (T_2_ versus T_1_, F = 13.32, p = 0.001, η^2^ = 0.232), (Fig 2). Pairwise comparison showed that the COTR group selected the speed significantly higher than the TR group at 4 weeks follow-up (T_2_), (t = -3.05, df = 45, p = 0.004), (Table 2, Fig 2). The self-selected speed at T_2_ was not different to baseline T_0_ in the TR group.

Concomitant heart rate measurements at self-selected speeds showed that cardiovascular strain was selected on a higher level after training (T1) compared with baseline (T0) (Fig 2, Table 2); ANCOVA with baseline heart rate as covariate reported a main effect of time (F = 8.67, df = 2, p = 0.001, η^2^ = 0.292), where contrasts revealed that the heart rate was a significant higher at post-training compared with baseline (T1 versus T0: F = 7.16, df = 1, p = 0.01, η^2^ = 0.143) and with 4 weeks follow-up (T2 versus T1: F = 6.42, df = 1, η^2^ = 0.130) in both groups. Moreover, a significant interaction of time x group was reported (F = 5.43, df = 2, p = 0.008, η^2^ = 0.205), where the interaction was based on the difference in change of heart rate between T2 and T1 between groups (F = 6.55, df = 1, p = 0.014, η^2^ = 0.132), showing that COTR group maintained a higher cardiovascular strain at 4 weeks follow-up compared with TR, which is consistent with the conditioning effect in self-selected speed (COTR).

Furthermore, ANCOVA with baseline as a covariate showed, that RPE levels (Table 2, Fig 2) at self-selected speed were increased after training (main effect of time: F = 17.58, df = 2, p<0.0001, η^2^ = 0.456; contrast T2 versus T1: F = 33.10, df = 1, p<0.0001, η^2^ = 0.435), as expected from higher cardiovascular strain at a higher speed. Moreover, a significant interaction of group x time was reported (F = 3.78, df = 2, p = 0.031, η^2^ = 0.153), where the interaction was significant between T1 and TO (F = 5.38, df = 1, p = 0.025, η^2^ = 0.111). The increase in RPE was stronger in the COTR group than the TR group between baseline and post-training. Pairwise comparison of RPE levels within groups showed that RPE levels between baseline and 4 weeks follow-up were not different in TR group, while 4 weeks follow-up RPE was significantly elevated compared with baseline in COTR group (t = -3.59, df = 21, p = 0.002); RPE was not returning to baseline at T2 in this group. To exclude any possible alteration in effort perception induced by training or conditioning we normalized RPE values on heart rate, assuming that cardiovascular strain would be the main driver of RPE during running at a self-selected speed. Mixed model ANOVA reported no significant main effects of time or group, and no interaction of time x group (not shown), supporting the assumption of unaltered effort perception.

### Effects of training and conditioning on reward discounting

Computer-based assessments of reward discounting of money, food, and exercise at baseline showed that the decay constant (k) for money (k_m_) was significantly lower than k for food (k_fo_) and exercise (k_ex_) across groups (k-values were log-transformed due to skewed distribution; repeated measure ANOVA: F = 69.96, df = 2, p<0.0001, η^2^ = 0.714; contrast k_m_ versus k_ex_: F = 130.2, df = 1, p<0.0001, η^2^ = 0.696); no significant difference between k_fo_ and k_ex_ was found. Outcomes demonstrate that exercise was discounted faster than money, like a non-transferrable reward, similar to food (Table 3).

**Table 3:** DD constants (k) of money (m), exercise (ex), and food (fo).

	Mean STD (±)	MED, (25^th^| 75^th^ PCTL)	R^2^ mean STD (±)
No Treatment (NTR) (N = 10)			
k_m_—T_0_	0.015 (0.025)	0.006 (0.003|0.014)	0.75 (0.15)
k_ex_—T_0_	0.076 (0.025)	0.079 (0.054|0.087)	0.79 (0.12)
k_ex_−T_1_	0.073 (0.026) #	0.082 (0.039|0.094)	0.75 (0.13)
k_fo_−T_0_	0.105 (0.085)	0.065 (0.043|0.155)	0.87 (0.09)
k_fo_−T_1_	0.122 (0.103)	0.086 (0.052|0.161)	0.86 (0.07)
Training (TR) (N = 24)			
k_m_—T_0_	0.049 (0.064)	0.029 (0.011|0.048)	0.87 (0.08)
k_ex_—T_0_	0.307 (0.296)	0.194 (0.080|0.550)	0.82 (0.10)
k_ex_−T_1_	0.131 (0.144)*	0.068 (0.033|0.163)	0.80 (0.11)
k_ex_−T_2_	0.238 (0.191)	0.159 (0.086|0.315)	0.88 (0.10)
k_fo_—T_0_	0.368 (0.404)	0.244 (0.095|0.456)	0.84 (0.10)
k_fo_−T_1_	0.359 (0.384)	0.226 (0.079|0.480)	0.85 (0.11)
k_fo_−T_2_	0.307 (0.354)	0.108 (0.088|0.481)	0.82 (0.10)
Training plus conditioning (COTR) (N = 24)			
k_m_—T_0_	0.0421 (0.062)	0.013 (0.007|0.045)	0.83 (0.13)
k_ex_—T_0_	0.326 (0.291)	0.145 (0.092|0.579)	0.88 (0.08)
k_ex_−T_1_	0.144 (0.181)*	0.055 (0.030|0.195)	0.84 (0.08)
k_ex_−T_2_	0.263 (0.255)	0.179 (0.064|0.392)	0.85 (0.11)
k_fo_—T_0_	0.321 (0.257)	0.265 (0.147|0.459)	0.86 (0.10)
k_fo_−T_1_	0.260 (0.239)	0.202 (0.062|0.396)	0.84 (0.11)
k_fo_−T_2_	0.302 (0.246)	0.280 (0.085|0.465)	0.81 (0.08)

*Significant effect of training, p<0.05

#significant interaction of group x time (NTR, TR), p<0.05. MED, median; PCTL, percentiles; STD, standard deviation.

To assess the hypothesized specific exercise training effect on discounting rates of exercise, ANOVAs of change between baseline and post-training values were performed on the log-transformed decay constants of k_ex_ and k_fo_ using the data of NTR and TR groups. This method was preferred to a mixed model ANOVA due to significantly lower levels of discounting rate constants in the NTR group compared with TR group at baseline (t-test: k_ex_: t = 3.02, df = 31.39, p = 0.0002; k_fo_: t = 2.68, df = 25.52, p = 0.013). Results revealed a significant difference between TR and NTR groups in the change of K_ex_ from baseline to after training and no-training periods, respectively (Δk_ex_: F = 13.80, df = 1, p = 0.001); k_ex_ was significantly reduced after training while k_ex_ in the NTR group was unaltered (Table 3). Moreover, this effect of exercise training was specific to k_ex_; the changes in k_fo_ from baseline to after training/no-training period were not significantly different between groups, and no change over time was reported within groups. Consequently, these results show that exercise training reduced discounting rates of exercise specifically; no effect on k_fo_, and discounting of both, exercise and food rewards, were not affected by time (no change in NTR group).

**Fig 3:**
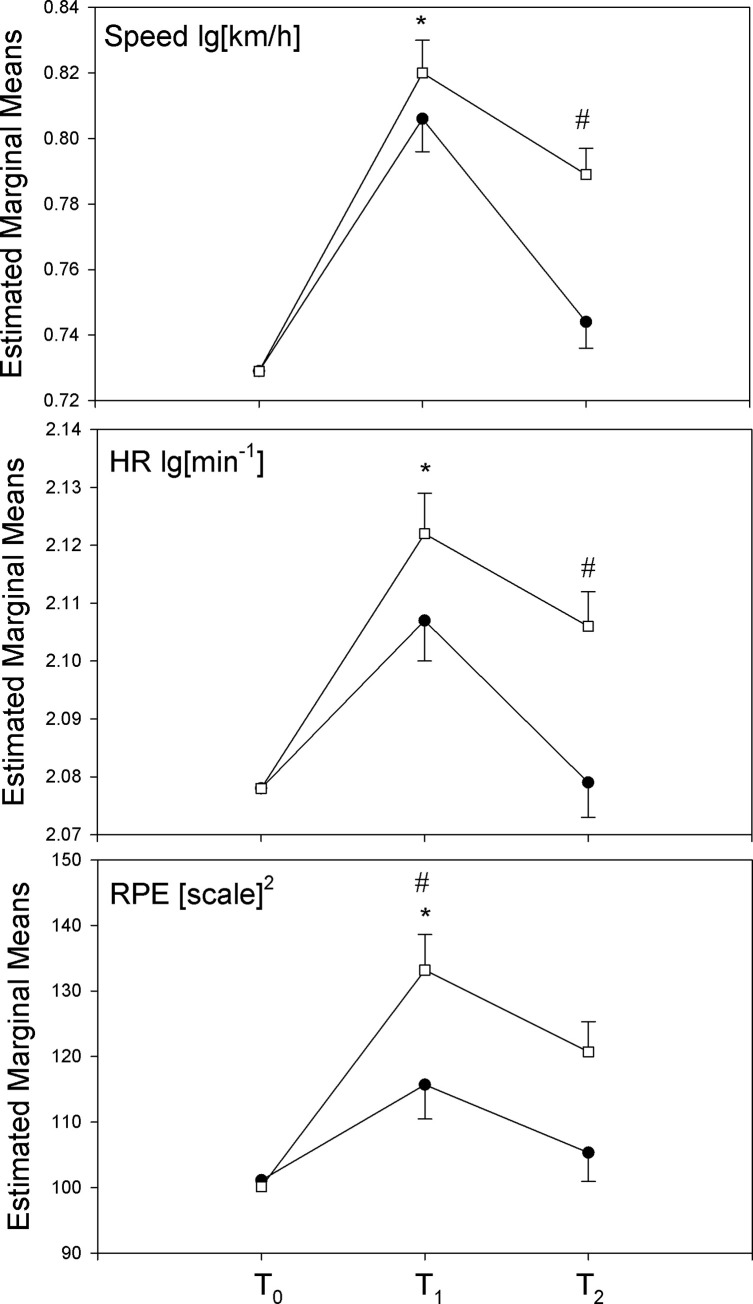
Log-transformed k_ex_ and k_fo_ changes over the three time points; baseline (T_0_), post-training (T_1_), and follow-up (T2) for the training (TR) and conditioning plus training (COTR) groups. Open boxes COTR; closed boxes TR. *Significant effect of training, p<0.05; the figure shows means and SE. Data are depicted and used in the form of meeting statistical test assumptions. Untransformed data are presented in Table 3.

For the hypothesis of an influence of EC on discounting rates of exercise, we used the data from the randomized control trial, TR, and COTR groups. Groups revealed no significant difference of k_ex_ and k_fo_ at baseline and mixed model ANOVA of log-transformed data over three time points (baseline–T_0_; post-exercise–T_1_; 4 weeks follow-up–T_2_) was performed. Results (Table 3, Fig 3) showed a main effect of time (F = 24.56, df = 2, p<0.0001, η^2^ = 0.522), where the post-training k_ex_ values (T_1_) were significantly reduced compared with baseline (T_0_) (contrast T_1_ versus T_0_: F = 45.78, df = 1, p<0.0001, η^2^ = 0.499). Moreover, contrasts revealed that k_ex_ values returned towards baseline levels in both groups at 4 weeks follow-up (T_2_ versus T_1_; F = 27.94, df = 1, p<0.0001, η^2^ = 0.378), with no significant differences between baseline and follow-up. Moreover, no significant effects of group and no interaction of group x time were found. In contrast, k_fo_ values were not affected by training or conditioning (Table 3, Fig 3), no significant effects of time, group, and interaction were reported, revealing stable levels of food reward discounting over time in both groups.

### Multiple regression analysis

We assumed that the self-selection of exercise intensity results from perceptual and evaluative processes, involving training status, effort perception, and evaluation of exercise as reward and cost. Consequently, we used the changes in variables training adaptation (HR/speed), exercise delay discounting (k_ex_), effort perception (RPE), as well as evaluative conditioning (group), for the explanation of variance in exercise intensity change over the study time points. We performed multiple regression analysis using the enter method adding the alterations of the former parameters from baseline to either post-training or follow-up as explanatory variables. In addition, we used age as a further explanatory variable because of the known age-dependent response to training. The first model (Table 4) analyzed the period from baseline to post-training (T_0_ to T_1_). The model explained about 60% of the variance of self-selected speed alterations, where the alteration in HR/speed, as a measure of training adaptation, explained most of the variance (beta = -0.600, p<0.0001), next to the alteration in RPE (beta = 0.396, p = 0.001), exercise discounting change and group did not contribute to the model. Higher increases in self-selected speed over this period were associated with stronger training adaptation (reduction in HR/speed) and larger effort acceptance (higher RPE).

**Table 4:** Multiple regression analysis of self-selected speed changes.

Model 1			
Speed Change T_1_-T_0_ (TR, COTR; n = 45)			
R^2^ = 0.59	F = 11.34	P<0.0001	
Variable	Beta	t	p
Age	-0.102	-0.991	0.328
Group	0.078	0.719	0.476
ΔHR/Speed T_1_-T_0_	-0.600	-5.81	<0.0001
Δk_ex_-T_1_-T_0_	-0.034	-0.332	0.741
ΔRPE T_1_-T_0_	0.396	3.67	0.001
Model 2			
Speed Change T_2_-T_0_ (TR, COTR; n = 46)			
R^2^ = 0.76	F = 25.23	P<0.0001	
Variable	Beta	t	p
Age	-0.362	-4.46	<0.0001
Group	0.428	5.25	<0.0001
ΔHR/Speed T_2_-T_0_	-0.487	-5.87	<0.0001
Δk_ex_-T_2_-T_0_	0.025	0.32	0.752
ΔRPE T_2_-T_0_	0.134	1.59	0.119

RPE, rate of perceived exertion/effort; HR, heart rate; Δ, difference.

For the self-selected speed alterations from baseline to follow-up (T_0_ to T_2_), the model (Table 4, model 2) explained about 76% of the variance; the significant variables were the grouping variable, showing the influence of conditioning (beta = 0.428, p<0.0001), and the alteration of HR/speed (beta = -0.487, p<0.0001), next to age. Higher speed was therefore selected under influence of EC and better training effect (HR/Speed) over this period.

### Effort model application

To integrate our findings into a concept which entails perceptual and evaluative parameters which seemed to explain exercise intensity selection, we suggested that choices have been made according to effort discounting models [25, 65, 66]. To test this, we used the hyperbolic effort discounting model Vp = M/(1+kC) [63], where the subjective value (Vp) is a function of the reward value (M) and perceived costs (C) in connection with the constant k. For our exercise intensity question, we assumed that participants’ pleasantness scores (Feeling Scale (FS), see Table 3), recorded while selecting their self-selected speed, would be a measure of the subjective value (Vp) of the exercise. Moreover, we assumed that the rate of perceived exertion (RPE) given at self-selected speed would be a measure of perceived costs (C), and the parameter HR/Speed a measure that would determine the reward value (M) perceived during exercise. This reward value could be influenced by expected rewards through learning. Using the data from time points T_0_, T_1_, and T_2_, assuming, that if the model is valid for the combination of data, the Vp data from the least-squares fit would not be significantly different from the measured FS values (Vp) data. Also, we expected that the k values of at T_0_ and T_1_ and T_2_ TR (training only group) would be on the same level, while the k value at T_2_ COTR of the conditioning group should be hugely affected by the added conditioned reward, which is not accounted for in the model. Because k is connected to costs, an unaccounted reward value (conditioning) would need to reduce the cost term by reducing k to adjust to the subjective value measured. Firstly, fitting of the hyperbolic model to data at baseline (T_0_), after training (T_1_), and follow up (T_2_) produced Vp values, which were not significantly different from the measured FS values (Table 4) (n = 48; T_0_: Z = -0.862, p = 0.389; T_1_: Z = -1.005, p = 0.315; T_2_: -1.005, p = 0.315), suggesting an appropriate representation of the data by the model. Moreover, when groups were separately analyzed at follow-up point (T_2_), based on the significant effects of conditioning on speed and effort selection (see Table 5), model fitting was improved (n = 24; 2-TR: Z = -0629, p = 0.530; T2-COTR: Z = -0.743, p = 0.458). k values of the fitted models reveal that ks are consistent over the periods of baseline, after training and follow up (T_2_, TR only): k-T_0_ = 0.204; k-T_1_ = 0.204; k-T_2_ (TR) = 0.212. However, k-T_2_ (COTR) = 0.042, revealed that the k value in the conditioning group (COTR) was adjusted five times lower to accommodate for the unaccounted reward value from the conditioning effect. The effort discounting model, therefore, fitted the observed data of our experiments reasonably and represents a possible explanation for the decision-making process.

**Table 5:** Hyperbolic effort model fit.

	Mean	STD	Wilcoxon Sign test	k
V_p_ (norm. FS-T_0_)	0.854	0.192		
V_p_−T_0_ model (n = 48)	0.823	0.134	Z = -0.862; p = 0.389	0.204
V_p_ (norm. FS-T_1_)	0.867	0.181		
V_p_−T_1_ model (n = 48)	0.848	0.119	Z = -1.005; p = 0.315	0.204
V_p_ (norm. FS-T_2_)	0.949	0.182		
V_p_−T_2_ model (n = 48)	0.922	0.136	Z = -1.005; p = 0.315	0.126
V_p_ (norm. FS-T_2_)TR	0.936	0.183		
V_p_−T_2_ model TR (n = 24)	0.908	0.120	Z = -0.625; p = 0.530	0.212
V_p_ (norm. FS-T_2_) COTR	0.963	0.180		
V_p_−T_2_ model COTR (n = 24)	0.940	0.134	Z = -0.743; p = 0.458	0.042

V_p_, normalized subjective feeling scale values; k, effort discounting constant.

## Discussion

### Alterations in self-selected speed: Effects of training and evaluative conditioning

We hypothesized that exercise training would lead to a transient increase of self-selected speed due to transient physiological adaptations and a decline at the follow-up. Evaluative conditioning would increase self-selected speed selection due to the integration of the reward into the visceral reward of exercising. The hypotheses were driven by the assumption that self-selected exercise intensity could be perceived as a reward at an individual level of cardiovascular strain influenced by transient training adaption and balanced against the perceived effort at a given intensity.

Indeed, self-selected speed was significantly increased after training (TR), no changes were detected in the control group (NTR) over time, showing that the training effect on self-selected speed was specific to training. Multiple regression analysis (Table 4) showed that the speed changes could be foremost attributed to training adaptation i.e. changes in heart rate per speed (HR/Speed) and RPE score changes. HR per running speed at submaximal levels has a linear association over a wide range of intensity and has been used for monitoring training and as a predictor of endurance performance in connection with cardiovascular fitness [67, 68]. Alterations in HR/Speed are connected to physiological adaptations to training enabling lower heart rate at a set speed after training [68]; however, the transient nature of those is seen at follow-up four weeks after training. Adaptation to aerobic training and detraining concerning heart rate changes are commonly observed and an expected outcome [69, 70].

To understand the specific selection for a speed and why training, as well as conditioning led to an alteration of it, we assumed that the selection of exercise intensity would follow models generally suggested for choice decisions that include costs and rewards [25, 27, 71]. Indeed, most behavioural models assume that the subjective value of a utility is a function of rewards and costs [27, 63, 72, 73]. Distinct choice paradigms where subjects work for an external reward with varied imagined or received rewards (i.e. money) have shown that a reward value is discounted against effort or work, resulting in a subjective value for the rewarding utility [25, 65, 66]. In confirmation, many animal studies showed that rewards and costs are similarly discounted in behavioural choices as well [25, 26, 28, 30]. In this regard, various models are suggested from hyperbolic [63], sigmoidal [74] to parabolic [27]; however, the principal rule applies that effort carries a negative value which is used as a reference against a reward is evaluated [25].

If we assume that adjustment of self-selected speed is a function of cost-reward, it is conceivable that training could have either reduced the perceived costs for a set speed or increased the perceived reward. Indeed, the higher the reduction of HR/Speed after training was for an individual, the higher the speed was selected, which could be an indication for perceived cost reduction. However, subjects selected an increased speed with even higher RPE scores, where RPE is undoubtedly a measure of costs, which weakens the former argument. RPE is often associated with individuals’ heart rate in aerobic exercise [75] and scales are partially attuned to heart rate levels i.e. scale 6–20 [76]. However, the integration of cardiovascular response (heart rate) into effort level perception does not exclude the possibility that fitness, i.e. heart rate per workload, could determine the reward perceived at a certain workload. Indeed, heart rate is not always associated with effort perception, attention allocation influences the association, and at higher heart rates with increased workloads, the attention shifts more closely towards physiological sensation i.e. heart rate [77]. Accordingly, we propose that HR/Speed, i.e. cardiovascular fitness, might determine the intensity of workload an individual selects based on its properties of limiting what can be perceived as rewarding. In our paradigm, subjects do not discount effort against an external reward but against a visceral reward made available by exercising at a distinct level of cardiovascular strain.

Additional support for this assumption of a modifiable reward in exercise linked to cardiovascular strain comes from our experiment using EC. We performed EC using our new paradigm, where the receipt of the sweet solution as a reward was associated with elevation of heart rate during exercise training. We hypothesized that EC would increase self-selected speed after training and follow-up, assuming that the conditioned reward would be integrated into the exercise reward enabling higher speed selection with concomitant increase in RPE. Indeed, the conditioning (COTR group) resulted in a significant increase in self-selected speed at follow-up compared with the training group (TR), while the former training effect on speed selection was not maintained over the follow-up period in the TR group. In the conditioning group (COTR) self-selected speed was preserved on a significantly higher level (about 1km/hr higher than baseline); the higher self-selected speed in COTR was associated with significantly higher heart rate and RPE than in TR group. However, EC effect was not significant directly after the training (T_1_), which could be due to the strong training effect on speed selection at this time point.

In terms of the conditioning process, there is no doubt about the rewarding nature of sweetness in humans [78, 79], and our participants were tested in a taste test about the pleasantness of the tastant used. Moreover, the brain areas known to be activated and concerned with reward are heavily activated in response to sweetness [80], even with non-caloric sweeteners [78, 79]. The selection of higher speed, cardiovascular strain (HR), and RPE in the conditioning group (COTR), shows that higher costs are chosen, which can only be explained by the integration of the sweet reward into the processes relevant for the speed selection. If exercise intensity would only be selected based on minimizing costs of ‘travel’, the EC would be without effect due to the lack of principal integration of rewards into the selection of speed.

Evaluative conditioning paradigms in humans usually used visual representations of an object or behaviour for the pairing with the unconditioned stimulus [37]. In connection with exercise, there are only two studies that applied EC to exercise behaviour using visual stimuli for the exercise representation and the unconditioned stimulus [41, 42]; however, only the study by Antoniewicz and Brand [42] observed acute exercise intensity changes after the EC procedure. To our knowledge, our study is the first study that has used the pairing of a physiological parameter during the performance of the behaviour with a primary reinforcer as UCS. Moreover, we associated the intensity of a tastant reward with the intensity of physical strain (heart rate), to direct the effect of the EC towards the selection of higher intensity in our self-selected exercise task. The use of primary reinforcers as unconditioned stimulus, tastants (rewarding and aversive), in connection with visual representations of food items and other objects, modifying food choices or implicit attitude towards selected food items, has been used before but only in a limited number of studies [81, 82].

To integrate our findings of training and conditioning, we further explored our data using the hyperbolic effort discounting model to calculate effort discounting constant k [63]. Outcomes revealed that using the feeling scale values as a measure of subjective value, the RPE values as costs, and the HR/Speed values as a determinant of reward, the model predicted the feeling scores successfully at all three study time points (Table 5). Indeed, the fitting produced a consistent k value for effort discounting at baseline, after training, and at follow-up for the TR group, while the k value of the COTR deviated strongly at the follow-up time point, where significant conditioning effects were detected for speed and RPE. The five times smaller k value for the COTR group at follow-up could be explained by an additional reward which was not imputed in the equation, consistent with our assumption that EC added a reward apart from the one determined by HR/Speed values. Usually, paradigms in effort discounting use an external reward to be gained by various degrees of workload [25, 29, 66, 74, 83]; and k values within a similar range of our data have been reported [29, 84]. However, in our case the reward is the self-selected exercise itself, which is determined by cardiovascular fitness (i.e. HR/Speed.) of the individual and the conditioned reward; concurrently, the effort invested (RPE) is adjusted to the paradigm’s demand of maximization of pleasantness (i.e. subjective value). Consequently, subjects do not adjust the speed to the lowest possible effort (RPE) or maintain the level. In our opinion, this interpretation makes also evolutional sense if we integrate the idea of foraging and hunting into the interpretation; humans with elevated physical fitness would ‘travel’ larger distances, enabling them to increase the probability of success in their foraging/hunting. Selection of speed or workload intensity would be selected as rewarding based on the specific capacity of an individual, apart from the exercise itself. Our conditioning experiment also suggests that learned/predicted rewards can be integrated with visceral rewards (i.e. fitness dependent). It is possible that reported associations in volume of elevated exercise intensity with intrinsic motivation could be indirectly attributed to this mechanism [85, 86].

### Alterations in delay discounting: Effects of training and evaluative conditioning

Our study shows that taking part in exercise training shifted the choice preference for exercise towards delayed option in the discounting paradigm; k_ex_ was significantly reduced in the training group (TR) but remained unaltered in the no-training group (NTR) after the three weeks intervention period. This effect was specific for exercise discounting; no alterations in food discounting were detected over time in both groups. Moreover, the effect on exercise discounting was not sustained after the four weeks follow-up period, where participants stopped training and returned to their habitual physical activity; k_ex_ returned to baseline at follow-up. Moreover, k_ex_ values have not been influenced by EC, no interaction of group and time was reported, while a reduction was expected due to a magnitude effect that would reduce k values [49]. However, there is a caveat for this expectation; the paradigm asked people to optimize the pleasantness, based on the feeling scale (optimizing subjective value), by adjusting the speed. Concurrently, participants perceived the same pleasantness, producing matching subjective values over time and groups. Therefore, the added reward by conditioning might not have revealed itself in the discounting paradigm, while it was apparent in the selection of speed at the follow-up time point. Moreover, propositions by Loewenstein [35] are referring to the problem of using past and likely future visceral factors for decision making. In general, visceral factors are underestimated in their capability for influencing behaviour if it comes to decision making regards cognitive evaluation and planning of future behaviour, as well as the actual impact for a behaviour. It seems, that the information of a visceral change towards a higher reward, which resulted in higher speed selection was not included in the processing of information for the delay discounting of exercise. Indeed, if the visceral changes in exercise experience would be integrated, we would detect an association between k_ex_ changes and speed changes; however, the alterations in k_ex_ were not correlated with the speed changes (not shown). This again suggests that the process for the actual selection of speed was not driven by the same factors as the delay discounting of the exercise.

We could formerly show that motivation towards delayed extrinsic goals, i.e. related to health and fitness, was associated with k_ex_ [31]. However, context-specific valuation could play a dominant role for the alteration of k_ex_ after training i.e. contextual relevance of delayed exercise training goals, which is also supported by the return to baseline k_ex_ levels at follow-up, where the contextualization of delayed goals is expected to decline. In agreement with this interpretation, discounting was shown to be context-sensitive in gamblers where k values were higher in a gambling environment than in a neutral environment [87]. When a situation or state is not directly experienced anymore, it turns to be more psychologically distant and would need more abstract cognitive representation [88]. The mental representation of delayed exercise training outcomes and goals might be more psychologically distant and decontextualized with emerging time distance to the training period. Alterations in temporal discounting by manipulations of construal levels (concrete or abstract) and psychological distance has been shown experimentally [89]. Further support for this interpretation, is that discounting alterations were specific for exercise and not seen in food discounting, revealing no generalized effect on discounting.

Our study has limitations; particularly, our paradigm limits the generalization to other exercise types which might be connected to other rewarding stimuli (group exercises, competitions, etc.). Moreover, self-selection of intensity might be limited in many team and competitive sports, therefore reducing the relevance of our findings in those areas. In addition, our participants had already a high exercise motivation at baseline, limiting the expansion of finding to groups with low motivation i.e. sedentary. In addition, we did not investigate sex differences in this study; sex differences in self-selected speed, RPE, and avHR were not detected, while slightly lower feeling scale values were detected for women (data not shown).

In conclusion, our study suggests that self-selected exercise can be perceived and evaluated as a reward. However, the intensity of exercise and exercise as a modality are differentially evaluated in the context of delay and effort discounting. The self-selected intensity of exercise, which can be perceived as rewarding, is determined by cardiovascular fitness, and learned rewards, and is discounted against perceived effort. However, delay discounting of self-selected exercise as a modality seemed to be strongly influenced by contextual factors and exercise motivation.

## Supporting information

S1 File Supplementary information. (DOCX)

S2 File Study raw data. (XLSX)

## References

[1] KESANIEMI YA, DANFORTH E, Jensen MD, Kopelman PG, Lefèbvre P, Reeder BA. Dose-response issues concerning physical activity and health: an evidence-based symposium. Medicine & Science in Sports & Exercise. 2001;33(6):S351–S8. 11427759 10.1097/00005768-200106001-00003

[2] Sofi F, Valecchi D, Bacci D, Abbate R, Gensini G, Casini A, et al. Physical activity and risk of cognitive decline: a meta‐analysis of prospective studies. Journal of internal medicine. 2011;269(1):107–17. doi: 10.1111/j.1365-2796.2010.02281.x 20831630

[3] Chapman JJ, Fraser SJ, Brown WJ, Burton NW. Physical activity preferences, motivators, barriers and attitudes of adults with mental illness. Journal of Mental Health. 2016;25(5):448–54. doi: 10.3109/09638237.2016.1167847 27049695

[4] Brownson RC, Boehmer TK, Luke DA. Declining rates of physical activity in the United States: what are the contributors? Annu Rev Public Health. 2005;26:421–43. doi: 10.1146/annurev.publhealth.26.021304.144437 15760296

[5] Hallal PC, Bauman AE, Heath GW, Kohl 3rd HW, Lee I-M, Pratt M. Physical activity: more of the same is not enough. The Lancet. 2012;380(9838):190–1. 10.1016/S0140-6736(12)61027-7 22818932

[6] Farooq M, Sazonov E, editors. Real time monitoring and recognition of eating and physical activity with a wearable device connected to the eyeglass. 2017 Eleventh International Conference on Sensing Technology (ICST); 2017: IEEE.

[7] Swain DP, Franklin BA. Comparison of cardioprotective benefits of vigorous versus moderate intensity aerobic exercise. The American journal of cardiology. 2006;97(1):141–7. doi: 10.1016/j.amjcard.2005.07.130 16377300

[8] van Waart H, Stuiver MM, van Harten WH, Geleijn E, Kieffer JM, Buffart LM, et al. Effect of low-intensity physical activity and moderate-to high-intensity physical exercise during adjuvant chemotherapy on physical fitness, fatigue, and chemotherapy completion rates: results of the PACES randomized clinical trial. Journal of Clinical Oncology. 2015;33(17):1918–27. doi: 10.1200/JCO.2014.59.1081 25918291

[9] Dishman RK, Sallis JF, Orenstein DR. The determinants of physical activity and exercise. Public health reports. 1985;100(2):158. 3920714 PMC1424729

[10] Duncan MJ, Badland HM, Mummery WK. Physical activity levels by occupational category in non-metropolitan Australian adults. Journal of Physical Activity and Health. 2010;7(6):718–23. doi: 10.1123/jpah.7.6.718 21088301

[11] Ingledew DK, Markland D, Ferguson E. Three levels of exercise motivation. Applied psychology: health and well‐being. 2009;1(3):336–55.

[12] Egli T, Bland HW, Melton BF, Czech DR. Influence of age, sex, and race on college students’ exercise motivation of physical activity. Journal of American college health. 2011;59(5):399–406. doi: 10.1080/07448481.2010.513074 21500059

[13] Deci EL, Ryan RM. Motivation and self-determination in human behavior. NY: Plenum Publishing Co. 1985. doi: 10.1097/00007691-198512000-00010

[14] Cheval B, Radel R, Neva JL, Boyd LA, Swinnen SP, Sander D, et al. Behavioral and neural evidence of the rewarding value of exercise behaviors: a systematic review. Sports Medicine. 2018;48(6):1389–404. doi: 10.1007/s40279-018-0898-0 29556981

[15] Frazao DT, de Farias Junior LF, Dantas T, Krinski K, Elsangedy HM, Prestes J, et al. Correction: feeling of pleasure to high-intensity interval exercise is dependent of the number of work bouts and physical activity status. PloS one. 2016;11(4):e0153986. doi: 10.1371/journal.pone.0153986 27077908 PMC4831771

[16] Bartlett JD, Close GL, MacLaren DP, Gregson W, Drust B, Morton JP. High-intensity interval running is perceived to be more enjoyable than moderate-intensity continuous exercise: implications for exercise adherence. Journal of sports sciences. 2011;29(6):547–53. doi: 10.1080/02640414.2010.545427 21360405

[17] Ekkekakis P, Hall EE, Petruzzello SJ. Some like it vigorous: Measuring individual differences in the preference for and tolerance of exercise intensity. Journal of Sport and Exercise Psychology. 2005;27(3):350–74.

[18] Williams DM, Dunsiger S, Ciccolo JT, Lewis BA, Albrecht AE, Marcus BH. Acute affective response to a moderate-intensity exercise stimulus predicts physical activity participation 6 and 12 months later. Psychology of sport and exercise. 2008;9(3):231–45. doi: 10.1016/j.psychsport.2007.04.002 18496608 PMC2390920

[19] Rose EA, Parfitt G. A quantitative analysis and qualitative explanation of the individual differences in affective responses to prescribed and self-selected exercise intensities. Journal of Sport and Exercise Psychology. 2007;29(3):281–309. doi: 10.1123/jsep.29.3.281 17876968

[20] Focht BC. Perceived exertion and training load during self-selected and imposed-intensity resistance exercise in untrained women. Journal of Strength and Conditioning Research. 2007;21(1):183. doi: 10.1519/00124278-200702000-00033 17313286

[21] Williams DM, Raynor HA. Disentangling the effects of choice and intensity on affective response to and preference for self-selected-versus imposed-intensity physical activity. Psychology of Sport and Exercise. 2013;14(5):767–75.

[22] Kivetz R. The effects of effort and intrinsic motivation on risky choice. Marketing Science. 2003;22(4):477–502.

[23] Phillips PE, Walton ME, Jhou TC. Calculating utility: preclinical evidence for cost–benefit analysis by mesolimbic dopamine. Psychopharmacology. 2007;191(3):483–95. doi: 10.1007/s00213-006-0626-6 17119929

[24] Rudebeck PH, Saunders RC, Lundgren DA, Murray EA. Specialized representations of value in the orbital and ventrolateral prefrontal cortex: desirability versus availability of outcomes. Neuron. 2017;95(5):1208–20. e5. doi: 10.1016/j.neuron.2017.07.042 28858621 PMC5600902

[25] Botvinick MM, Huffstetler S, McGuire JT. Effort discounting in human nucleus accumbens. Cognitive, Affective, & Behavioral Neuroscience. 2009;9(1):16–27. doi: 10.3758/CABN.9.1.16 19246324 PMC2744387

[26] Walton ME, Bannerman DM, Rushworth MF. The role of rat medial frontal cortex in effort-based decision making. Journal of Neuroscience. 2002;22(24):10996–1003. doi: 10.1523/JNEUROSCI.22-24-10996.2002 12486195 PMC6758435

[27] Hartmann MN, Hager OM, Tobler PN, Kaiser S. Parabolic discounting of monetary rewards by physical effort. Behavioural processes. 2013;100:192–6. doi: 10.1016/j.beproc.2013.09.014 24140077

[28] Stevens JR, Hallinan EV, Hauser MD. The ecology and evolution of patience in two New World monkeys. Biology Letters. 2005;1(2):223–6. doi: 10.1098/rsbl.2004.0285 17148172 PMC1626214

[29] Klein-Flügge MC, Kennerley SW, Saraiva AC, Penny WD, Bestmann S. Behavioral modeling of human choices reveals dissociable effects of physical effort and temporal delay on reward devaluation. PLoS computational biology. 2015;11(3). doi: 10.1371/journal.pcbi.1004116 25816114 PMC4376637

[30] Salamone JD, Cousins MS, Bucher S. Anhedonia or anergia? Effects of haloperidol and nucleus accumbens dopamine depletion on instrumental response selection in a T-maze cost/benefit procedure. Behavioural brain research. 1994;65(2):221–9. doi: 10.1016/0166-4328(94)90108-2 7718155

[31] Albelwi TA, Rogers RD, Kubis H-P. Exercise as a reward: Self-paced exercise perception and delay discounting in comparison with food and money. Physiology & behavior. 2019;199:333–42. 30529339 10.1016/j.physbeh.2018.12.004

[32] Kirby KN, Maraković NN. Delay-discounting probabilistic rewards: Rates decrease as amounts increase. Psychonomic bulletin & review. 1996;3(1):100–4. doi: 10.3758/BF03210748 24214810

[33] Tesch AD, Sanfey AG. Models and methods in delay discounting. Annals of the New York Academy of Sciences. 2008;1128(1):90–4. doi: 10.1196/annals.1399.010 18469217

[34] McClure SM, Laibson DI, Loewenstein G, Cohen JD. Separate neural systems value immediate and delayed monetary rewards. Science. 2004;306(5695):503–7. doi: 10.1126/science.1100907 15486304

[35] Loewenstein G. Out of control: Visceral influences on behavior. Organizational behavior and human decision processes. 1996;65(3):272–92.

[36] De Houwer J, Thomas S, Baeyens F. Association learning of likes and dislikes: A review of 25 years of research on human evaluative conditioning. Psychological bulletin. 2001;127(6):853. doi: 10.1037/0033-2909.127.6.853 11726074

[37] De Houwer J. A conceptual and theoretical analysis of evaluative conditioning. The Spanish journal of psychology. 2007;10(2):230–41. doi: 10.1017/s1138741600006491 17992949

[38] Hofmann W, De Houwer J, Perugini M, Baeyens F, Crombez G. Evaluative conditioning in humans: a meta-analysis. Psychological bulletin. 2010;136(3):390. doi: 10.1037/a0018916 20438144

[39] Franken IH, Huijding J, Nijs IM, van Strien JW. Electrophysiology of appetitive taste and appetitive taste conditioning in humans. Biological Psychology. 2011;86(3):273–8. doi: 10.1016/j.biopsycho.2010.12.008 21187120

[40] Blechert J, Testa G, Georgii C, Klimesch W, Wilhelm F. The Pavlovian craver: Neural and experiential correlates of single trial naturalistic food conditioning in humans. Physiology & behavior. 2016;158:18–25. doi: 10.1016/j.physbeh.2016.02.028 26905451

[41] Martin L, Signorile JF, Kahn BE, Perkins AW, Ahn S, Perry AC. Improving Exercise Adherence and Physical Measures in English-Speaking Latina Women. Journal of racial and ethnic health disparities. 2015;2(4):517–26. doi: 10.1007/s40615-015-0100-4 26863558

[42] Antoniewicz F, Brand R. Learning to like exercising: Evaluative conditioning changes automatic evaluations of exercising and influences subsequent exercising behavior. Journal of Sport and Exercise Psychology. 2016;38(2):138–48. doi: 10.1123/jsep.2015-0125 27385674

[43] Lenoir M, Serre F, Cantin L, Ahmed SH. Intense sweetness surpasses cocaine reward. PloS one. 2007;2(8). doi: 10.1371/journal.pone.0000698 17668074 PMC1931610

[44] Cantin L, Lenoir M, Augier E, Vanhille N, Dubreucq S, Serre F, et al. Cocaine is low on the value ladder of rats: possible evidence for resilience to addiction. PloS one. 2010;5(7). doi: 10.1371/journal.pone.0011592 20676364 PMC2911372

[45] Huynh C, Fam J, Ahmed SH, Clemens KJ. Rats quit nicotine for a sweet reward following an extensive history of nicotine use. Addiction biology. 2017;22(1):142–51. doi: 10.1111/adb.12306 26374708

[46] Prévost C, Liljeholm M, Tyszka JM, O’Doherty JP. Neural correlates of specific and general Pavlovian-to-Instrumental Transfer within human amygdalar subregions: a high-resolution fMRI study. Journal of Neuroscience. 2012;32(24):8383–90. doi: 10.1523/JNEUROSCI.6237-11.2012 22699918 PMC6703659

[47] Madsen HB, Ahmed SH. Drug versus sweet reward: greater attraction to and preference for sweet versus drug cues. Addiction biology. 2015;20(3):433–44. doi: 10.1111/adb.12134 24602027

[48] Nolan TA, Caudle RM, Neubert JK. Effect of caloric and non-caloric sweet reward solutions on thermal facial operant conditioning. Behavioural brain research. 2011;216(2):723–5. doi: 10.1016/j.bbr.2010.08.023 20797411 PMC2981637

[49] Frederick S, Loewenstein G, O’donoghue T. Time discounting and time preference: A critical review. Journal of economic literature. 2002;40(2):351–401.

[50] Fogelholm M, Malmberg J, Suni J, Santtila M, Kyröläinen H, Mäntysaari M, et al. International physical activity questionnaire: Validity against fitness. Medicine & Science in Sports & Exercise. 2006;38(4):753–60. doi: 10.1249/01.mss.0000194075.16960.20 16679993

[51] Craig CL, Marshall AL, Sjöström M, Bauman AE, Booth ML, Ainsworth BE, et al. International physical activity questionnaire: 12-country reliability and validity. Medicine & science in sports & exercise. 2003;35(8):1381–95. doi: 10.1249/01.MSS.0000078924.61453.FB 12900694

[52] Markland D, Ingledew DK. The measurement of exercise motives: Factorial validity and invariance across gender of a revised Exercise Motivations Inventory. British Journal of Health Psychology. 1997;2(4):361–76.

[53] Hardy CJ, Rejeski WJ. Not what, but how one feels: the measurement of affect during exercise. Journal of sport and exercise psychology. 1989;11(3):304–17.

[54] Ekkekakis P, Lind E. Exercise does not feel the same when you are overweight: the impact of self-selected and imposed intensity on affect and exertion. International journal of obesity. 2006;30(4):652–60. doi: 10.1038/sj.ijo.0803052 16130028

[55] Borg G. Perceived exertion as an indicator of somatic stress. Scandinavian Journal of Rehabilitation Medicine. 1970;2(2):92–8. 5523831

[56] Richards JB, Zhang L, Mitchell SH, de Wit H. DELAY OR PROBABILITY DISCOUNTING IN A MODEL OF IMPULSIVE BEHAVIOR: EFFECT OF ALCOHOL. Journal of the Experimental Analysis of Behavior. 1999;71(2):121–43. doi: 10.1901/jeab.1999.71-121 10220927 PMC1284697

[57] Charlton SR, Fantino E. Commodity specific rates of temporal discounting: does metabolic function underlie differences in rates of discounting? Behavioural processes. 2008;77(3):334–42. doi: 10.1016/j.beproc.2007.08.002 17919848

[58] Rasmussen EB, Lawyer SR, Reilly W. Percent body fat is related to delay and probability discounting for food in humans. Behavioural processes. 2010;83(1):23–30. doi: 10.1016/j.beproc.2009.09.001 19744547

[59] Dodds M, Roland S, Edgar M, Thornhill M. Saliva A review of its role in maintaining oral health and preventing dental disease. Bdj Team. 2015;2:15123.

[60] Glautier S, Drummond C, Remington B. Alcohol as an unconditioned stimulus in human classical conditioning. Psychopharmacology. 1994;116(3):360–8. doi: 10.1007/BF02245341 7892428

[61] Tanaka H, Monahan KD, Seals DR. Age-predicted maximal heart rate revisited. Journal of the american college of cardiology. 2001;37(1):153–6. doi: 10.1016/s0735-1097(00)01054-8 11153730

[62] Van Breukelen GJ. ANCOVA versus change from baseline had more power in randomized studies and more bias in nonrandomized studies. Journal of clinical epidemiology. 2006;59(9):920–5. doi: 10.1016/j.jclinepi.2006.02.007 16895814

[63] Myerson J, Green L. Discounting of delayed rewards: Models of individual choice. Journal of the experimental analysis of behavior. 1995;64(3):263–76. doi: 10.1901/jeab.1995.64-263 16812772 PMC1350137

[64] Mazur JE. An adjusting procedure for studying delayed reinforcement. Commons, ML; Mazur, JE; Nevin, JA. 1987:55–73.

[65] Kurniawan IT, Seymour B, Talmi D, Yoshida W, Chater N, Dolan RJ. Choosing to make an effort: the role of striatum in signaling physical effort of a chosen action. Journal of neurophysiology. 2010;104(1):313–21. doi: 10.1152/jn.00027.2010 20463204 PMC2904211

[66] Białaszek W, Marcowski P, Ostaszewski P. Physical and cognitive effort discounting across different reward magnitudes: Tests of discounting models. PloS one. 2017;12(7). doi: 10.1371/journal.pone.0182353 28759631 PMC5536267

[67] Boudet G, Albuisson E, Bedu M, Chamoux A. Heart rate running speed relationships during exhaustive bouts in the laboratory. Canadian journal of applied physiology. 2004;29(6):731–42. doi: 10.1139/h04-047 15630146

[68] Vesterinen V, Häkkinen K, Hynynen E, Mikkola J, Hokka L, Nummela A. Heart rate variability in prediction of individual adaptation to endurance training in recreational endurance runners. Scandinavian journal of medicine & science in sports. 2013;23(2):171–80. doi: 10.1111/j.1600-0838.2011.01365.x 21812828

[69] Green JH, Cable N, Elms N. Heart rate and oxygen consumption during walking on land and in deep water. The Journal of sports medicine and physical fitness. 1990;30(1):49–52. 2366535

[70] Mujika I, Padilla S. Detraining: loss of training-induced physiological and performance adaptations. Part I. Sports Medicine. 2000;30(2):79–87. doi: 10.2165/00007256-200030020-00002 10966148

[71] Green L, Myerson J. A discounting framework for choice with delayed and probabilistic rewards. Psychological bulletin. 2004;130(5):769. doi: 10.1037/0033-2909.130.5.769 15367080 PMC1382186

[72] Samuelson PA. A note on measurement of utility. The review of economic studies. 1937;4(2):155–61.

[73] Zipf GK. Human behavior and the principle of least effort. 1949. 15405394

[74] Klein-Flügge MC, Kennerley SW, Friston K, Bestmann S. Neural signatures of value comparison in human cingulate cortex during decisions requiring an effort-reward trade-off. Journal of Neuroscience. 2016;36(39):10002–15. doi: 10.1523/JNEUROSCI.0292-16.2016 27683898 PMC5039251

[75] Eston R, Williams J. Reliability of ratings of perceived effort regulation of exercise intensity. British journal of sports medicine. 1988;22(4):153–5. doi: 10.1136/bjsm.22.4.153 3228684 PMC1478740

[76] Borg G. Borg’s perceived exertion and pain scales: Human kinetics; 1998.

[77] Tenenbaum G, Connolly CT. Attention allocation under varied workload and effort perception in rowers. Psychology of Sport and Exercise. 2008;9(5):704–17.

[78] Green E, Murphy C. Altered processing of sweet taste in the brain of diet soda drinkers. Physiology & behavior. 2012;107(4):560–7. doi: 10.1016/j.physbeh.2012.05.006 22583859 PMC3465626

[79] Griffioen-Roose S, Smeets PA, Weijzen PL, van Rijn I, van den Bosch I, de Graaf C. Effect of replacing sugar with non-caloric sweeteners in beverages on the reward value after repeated exposure. PLoS One. 2013;8(11). doi: 10.1371/journal.pone.0081924 24312382 PMC3842969

[80] Stice E, Burger KS, Yokum S. Relative ability of fat and sugar tastes to activate reward, gustatory, and somatosensory regions. The American journal of clinical nutrition. 2013;98(6):1377–84. doi: 10.3945/ajcn.113.069443 24132980 PMC3831532

[81] Andreatta M, Pauli P. Appetitive vs. aversive conditioning in humans. Frontiers in behavioral neuroscience. 2015;9:128. doi: 10.3389/fnbeh.2015.00128 26042011 PMC4436895

[82] Hensels I, Baines S. Changing ‘gut feelings’ about food: An evaluative conditioning effect on implicit food evaluations and food choice. Learning and Motivation. 2016;55:31–44.

[83] Klein-Fluegge M. Interaction of Decision Making and Action Planning in the Human Brain: UCL (University College London); 2014.

[84] Nishiyama R. Response effort discounts the subjective value of rewards. Behavioural Processes. 2014;107:175–7. doi: 10.1016/j.beproc.2014.08.002 25150069

[85] Martyn S, Simon JS, Tom L. Does Exercise Motivation Predict Engagement in Objectively Assessed Bouts of Moderate-Intensity Exercise?: A Self-Determination Theory Perspective. Journal of Sport and Exercise Psychology. 2008;30(4):337–52. doi: 10.1123/jsep.30.4.337 18723896

[86] Duncan LR, Hall CR, Wilson PM, Jenny O. Exercise motivation: a cross-sectional analysis examining its relationships with frequency, intensity, and duration of exercise. International Journal of Behavioral Nutrition and Physical Activity. 2010;7(1):7. doi: 10.1186/1479-5868-7-7 20181017 PMC2835648

[87] Dixon MR, Jacobs EA, Sanders S. Contextual control of delay discounting by pathological gamblers. Journal of applied behavior analysis. 2006;39(4):413–22. doi: 10.1901/jaba.2006.173-05 17236338 PMC1702333

[88] Trope Y, Liberman N. Temporal construal. Psychological review. 2003;110(3):403. doi: 10.1037/0033-295x.110.3.403 12885109

[89] Kim H, Schnall S, White MP. Similar psychological distance reduces temporal discounting. Personality and Social Psychology Bulletin. 2013;39(8):1005–16. doi: 10.1177/0146167213488214 23653066

