# Supplementary material for: Carrots for the donkey: Influence of evaluative conditioning and training on self-paced exercise intensity and delay discounting of exercise in healthy adults
Source: PLoS One. 2021 Oct 6;16(10):e0257953. doi: 10.1371/journal.pone.0257953 (PMC8494336; doi:10.1371/journal.pone.0257953)
Supplement: S1 File — (DOCX) [file pone.0257953.s001.docx]

**Supplementary Information**

**Self-Report Questionnaires**

1. Monetary Choice Questionnaire (MCQ)[1], is a 27-item questionnaire that assesses delay discounting of money using a set of choices between hypothetical monetary rewards of different magnitudes/values delivered at different delays; Cronbach’s alpha (CRα): 0.98 [2].
2. Food Craving Questionnaire-Trait (FCQ-T) [3] measures craving for foods, without confining them to certain categories, and covers behavioural, cognitive, and physiological aspects of cravings with 39-items. The overall CRα for the FCQ-T is 0.98 and subscale alphas ranges from 0.71 to 0.95 [3].
3. Barrat Impulsivity Scale (BIS II) [4] measures the personality/behavioural construct of impulsiveness based on scaling frequencies of common impulsive or non-impulsive behaviours and preferences. CRα was reported to be between 0.71 and 0.83 [5].
4. Physical Activity Readiness Questionnaire (PAR-Q)[6, 7] for assessment of potential health risks in association with physical activity participation.
5. Reward Responsiveness (RR) scale: a subscale of the Behavioural activation system (BAS) scales [8]. The RR scale measures the tendency to respond with heightened energy and positive affect when desired events are experienced or anticipated [9]. It includes a 5-item, self-report measure that assesses reward sensitivity using a 4-point Likert scale (1=very true for me, 4=very false for me). Internal consistencies of all BAS subscales were good (Cronbach's α ranged 0.68–0.79) [10].
6. General Positive and Negative Affect Schedule PANAS-G [11] to assess average mood state using two 10-item scales; CRα: 0.88 [12].

**Taste test**

To ensure the optimized pleasantness of the sweet conditioning solution, COTR subjects conducted a taste test. Perceived pleasantness was recorded using stepwise dilutions of the 100% sweet solutions (20mg sucralose plus 0.877g *Squincher Zero* powder /100ml) in randomized order, for potential adjustment of the most pleasant concentration. A nine-point hedonic scale “1 = disliked extremely”, “9 = liked extremely” [13] was used to rate the taste pleasantness for 5 sweet drink concentrations (100%, 75%, 50%, 25% and 12.5%). The participants were injected a volume of 5-ml for each concentration into the oral cavity with a syringe and asked to rate the taste pleasantness after rinsing mouth. Between the tested solutions, they took sips of water to flush out the remaining tastants. The concentration with the highest pleasantness rating was used as the highest sweet reward (100%) during conditioning; however, all participants rated the original 100% solution as most pleasant. Therefore, no further adjustments were performed.

**Drinks composition**

For the EC process, the following drink solution was used: SWEET SOLUTION: *Sqwincher Zero* Drink (Sqwincher, USA) with sucralose (MYPROTEIN.com) adjustment; the highest concentration of the sweet solution (100% sweetness) = 0.877g *Sqwincher Zero* powder plus 20mg sucralose per 100ml.

NEUTRAL SOLUTION: The neutral solution was used for (0% sweetness) based on an electrolyte drink (bulkpowders.co.uk) dissolved according to the manufacturer’s protocol.

Both solutions were used for loading 60 ml syringes for syringe pumps (see below); solutions were freshly prepared and used at room temperature (15-17Cº).

**Psychological self-report data**

Psychological self-report questionnaires revealed that participants perceived moderate cravings (FCQ-T: 130.6 (31.2)), scored moderate levels in the impulsivity questionnaire (BIS II: 69.6 (16.9)) and monetary choice questionnaire (MCQ) resulted in a median k_kirby_=0.01. The PANAS-PA and PANAS-NA reported more than medium scores for positive affect (38.0 (7.3)) and less than medium scores for negative affect (14.9 (4.7)). Participants reported a higher than medium rewards sensitivity (BASReward: 10.2 (3.7)). Comparison (Kruskal-Wallis test) of body characteristics and psychological self-report parameters reported no significant differences between groups, with exception of PANAS-PA (H:7.03, df:2, p=0.03), where follow-up tests (U-test) revealed significantly lower scores in the no-training group (NTR) compared with training (TR) and training plus conditioning groups (COTR), (U=64.5, p=0.035; U=50.0, p=0.008, respectively).

**S1 Table. Psychological self-report parameters**

|  | No Training (NTR) (N=10) | Training (TR) (N=24) | Training plus conditioning (COTR) (N=24) |
| --- | --- | --- | --- |
| MCQ-k_Kirby_ | 0.012 (0.019) | 0.020 (0.027) | 0.021 (0.035) |
| BIS II  (RG: 30-120) | 61.90 (13.38) | 74.92 (16.39) | 67.54 (17.56) |
| FCQ-T  (RG: 39-234) | 122.90 (27.21) | 140.50 (33.81) | 123.70 (28.39) |
| PANAS-G-PA  (RG: 10-50) | 32.60 (6.60) | 38.62 (6.86) | 39.54 (7.24) |
| PANAS-G-NA  (RG: 10-50) | 16.20 (4.37) | 14.08 (4.03) | 15.25 (5.38) |
| BASReward  (RG: 5-20)  High sensitivity = 5 | 9.90 (3.73) | 10.79 (4.55) | 9.75 (2.75) |

Mean and standard deviations of body characteristics and psychometric self-reports; RG, range of scores; PCT, percentage; abbreviations explained in text above.

**References**

1. Kirby KN, Petry NM, Bickel WK. Heroin addicts have higher discount rates for delayed rewards than non-drug-using controls. Journal of Experimental psychology: general. 1999;128(1):78.

2. Duckworth AL, Seligman ME. Self-discipline outdoes IQ in predicting academic performance of adolescents. Psychological science. 2005;16(12):939-44.

3. Cepeda-Benito A, Gleaves DH, Williams TL, Erath SA. The development and validation of the state and trait food-cravings questionnaires. Behavior therapy. 2000;31(1):151-73.

4. Patton JH, Stanford MS, Barratt ES. Factor structure of the Barratt impulsiveness scale. Journal of clinical psychology. 1995;51(6):768-74.

5. Vasconcelos AG, Malloy-Diniz L, Correa H. Systematic review of psychometric proprieties of Barratt Impulsiveness Scale Version 11 (BIS-11). Clinical Neuropsychiatry. 2012;9(2).

6. Chisholm D, Collis M, Kulak L, Davenport W, Gruber N. Physical activity readiness. BC Med J. 1975;17(2):375-8.

7. Shephard RJ. Physical activity, fitness and cardiovascular health: a brief counselling guide for older patients. CMAJ: Canadian Medical Association Journal. 1994;151(5):557.

8. Carver CS, White TL. Behavioral inhibition, behavioral activation, and affective responses to impending reward and punishment: the BIS/BAS scales. Journal of personality and social psychology. 1994;67(2):319.

9. Meyer B, Johnson SL, Winters R. Responsiveness to threat and incentive in bipolar disorder: Relations of the BIS/BAS scales with symptoms. Journal of psychopathology and behavioral assessment. 2001;23(3):133-43.

10. Markarian SA, Pickett SM, Deveson DF, Kanona BB. A model of BIS/BAS sensitivity, emotion regulation difficulties, and depression, anxiety, and stress symptoms in relation to sleep quality. Psychiatry research. 2013;210(1):281-6.

11. Mackinnon A, Jorm AF, Christensen H, Korten AE, Jacomb PA, Rodgers B. A short form of the Positive and Negative Affect Schedule: Evaluation of factorial validity and invariance across demographic variables in a community sample. Personality and Individual differences. 1999;27(3):405-16.

12. Carvalho HWd, Andreoli SB, Lara DR, Patrick CJ, Quintana MI, Bressan RA, et al. Structural validity and reliability of the Positive and Negative Affect Schedule (PANAS): evidence from a large Brazilian community sample. Brazilian Journal of Psychiatry. 2013;35(2):169-72.

13. Peryam DR, Pilgrim FJ. Hedonic scale method of measuring food preferences. Food technology. 1957.
